# “I feel like I am surviving the health care system”: understanding LGBTQ health in Nova Scotia, Canada

**DOI:** 10.1186/s12889-016-3675-8

**Published:** 2016-09-22

**Authors:** Emily Colpitts, Jacqueline Gahagan

**Affiliations:** Gender and Health Promotion Studies Unit, School of Health and Human Performance, Dalhousie University, Stairs House, 6230 South Street, P.O. Box 15000, Halifax, NS B3H 4R2 Canada

**Keywords:** LGBTQ, Health, Canada, Health promotion, Health research, Health measurement

## Abstract

**Background:**

Currently, there is a dearth of baseline data on the health of lesbian, gay, bisexual, transgender, and queer (LGBTQ) populations in the province of Nova Scotia, Canada. Historically, LGBTQ health research has tended to focus on individual-level health risks associated with poor health outcomes among these populations, which has served to obscure the ways in which they maintain their own health and wellness across the life course. As such, there is an urgent need to shift the focus of LGBTQ health research towards strengths-based perspectives that explore the complex and resilient ways in which LGBTQ populations promote their health.

**Methods:**

This paper discusses the findings of our recent scoping review as well as the qualitative data to emerge from community consultations aimed at developing strengths-based approaches to understanding and advancing LGBTQ pathways to health across Nova Scotia.

**Results:**

Our scoping review findings demonstrated the lack of strengths-based research on LGBTQ health in Nova Scotia. Specifically, the studies examined in our scoping review identified a number of health-promoting factors and a wide variety of measurement tools, some of which may prove useful for future strengths-based health research with LGBTQ populations. In addition, our community consultations revealed that many participants had negative experiences with health care systems and services in Nova Scotia. However, participants also shared a number of factors that contribute to LGBTQ health and suggestions for how LGBTQ pathways to health in Nova Scotia can be improved.

**Conclusions:**

There is an urgent need to conduct research on the health needs, lived experiences, and outcomes of LGBTQ populations in Nova Scotia to address gaps in our knowledge of their unique health needs. In moving forward, it is important that future health research take an intersectional, strengths-based perspective in an effort to highlight the factors that promote LGBTQ health and wellness across the life course, while taking into account the social determinants of health.

## Background

Currently, there is an absence of baseline data on the health of lesbian, gay, bisexual, transgender, and queer (LGBTQ) populations in the province of Nova Scotia, Canada. Studies from other regions of Canada (Ontario, British Columbia, and Quebec), as well as the United States and the United Kingdom suggest that the health of LGBTQ populations is worse than that of their heterosexual, cisgender age-matched peers [1, 2]. Given that Atlantic Canada, including Nova Scotia, tends to have worse health outcomes than other regions in Canada [3], the dearth of data specific to LGBTQ health in Nova Scotia is of particular concern. For example, in comparison with the national average, Statistics Canada data indicate that Nova Scotia has higher overall rates of obesity (60 % versus 52 %), arthritis (26 % versus 15 %), diabetes (8 % versus 6 %), high blood pressure (21 % versus 17 %), chronic obstructive pulmonary disease (COPD) (6 % versus 4 %), colon cancer (60 % versus 50 %), heavy drinking (20 % versus 17 %), lung cancer (54 % versus 45 %), and a lower rate of functional health (77 % versus 81 %) [4]. Although these data are important in advancing our understanding of the overall health conditions impacting the health of Nova Scotians, they do not specifically refer to the health of LGBTQ populations in Nova Scotia.

Conducting LGBTQ health research in Nova Scotia is critically important given that LGBTQ health needs have historically been understood through a heteronormative, gender-binary lens, which assumes that the health needs of LGBTQ populations are similar to those of their heterosexual, cisgender age-matched peers [2, 5, 6]. This heteronormative and gender-binary approach to LGBTQ health has effectively rendered the health needs and experiences of these populations invisible within mainstream health care systems, health data, and health policies [6, 7]. The invisibility or erasure [7] of LGBTQ populations and their specific health needs and lived experiences hinders the provision of evidence-based, culturally competent health care for these populations. Previous health research from projects conducted outside of Nova Scotia demonstrates that LGBTQ populations experience significant discrimination and stigma within health care systems based on the heteronormative and gender-binary framing of health [8, 9]. For example, Goins and Pye [9] found that the heteronormative and gender-binary language and structure of medical intake forms have the consequence of alienating LGBTQ populations. While the full impact of this form of invisibility or erasure on the health of LGBTQ populations in Nova Scotia is not well understood, a previous study on the experiences of queer and trans women in Nova Scotia found that participants experienced significant discomfort in their interactions with healthcare providers and expressed fear that they would be denied adequate health care based on their sexual orientation or gender identities [10].

The way in which health and wellness are defined has important implications for how research evidence is understood and reported. The Public Health Agency of Canada (PHAC) borrows its definition of health from the World Health Organization (WHO), which has long defined health as “a state of complete physical, social and mental well-being, and not merely the absence of disease or infirmity” [11]. The PHAC also recognizes that the health of individuals and populations is influenced by a variety of intersecting and overlapping determinants at both the individual and structural levels, including income and social status, social support networks, education and literacy, employment/working conditions, social environments, physical environments, personal health practices and coping skills, healthy child development, biology and genetic endowment, health services, gender, and culture [12]. Despite this recognition, public health policy and practice in Canada have traditionally focused on individual health and on interventions that target individual behaviour [6, 13]. The emphasis on individual-level health outcomes further obscures the ways in which structural determinants of health such as heteronormative health care systems and policies in Canada can negatively impact on health care access and uptake among LGBTQ populations [6].

Although PHAC does not recognize LGBTQ identity as a key social determinant of health [12], it is important to consider how sexual orientation and gender identity intersect with other determinants to shape the health of LGBTQ individuals. The concept of intersectionality is key to understanding health outcomes among LGBTQ populations in that health is determined by the complex interactions of LGBTQ identity with other factors such as race, socioeconomic status, age, social exclusion, employment, etc. [14, 15]. For example, previous research has demonstrated that LGBTQ populations experience higher rates of homelessness, social exclusion, poverty, and other negative determinants of health compared to their heterosexual, cisgender, age-matched peers [16–19]. Bowleg, Huang, Brooks, Black, and Burkholder contend, for example, that the health of black lesbians is affected by the ‘triple threat’ of racism, sexism, and heterosexism [20]. Further, social stigma, discrimination, and victimization have been found to not only have negative effects on physical and mental health [21], but also to affect rates of access to and uptake of preventative health screening programs and health care services among LGBTQ populations [6, 22–24]. According to the findings of an earlier study on lesbian and bisexual women’s experiences with family physicians in Nova Scotia, more than two-thirds of the 98 women interviewed reported encountering heterosexist assumptions and many women reported avoiding routine or preventative health care due to health care providers’ heteronormative assumptions [25]. As such, the overemphasis on individual-level health factors contributes to the invisibility or erasure of the health needs and experiences of LGBTQ populations by obscuring the unique social, structural, and systemic determinants that impact the health outcomes of these populations [26, 27].

The lack of health data on, and the resultant invisibility of, LGBTQ health needs and experiences in Nova Scotia highlights the importance of conducting research focused specifically on these topics. The long history of health research approaches that have tended to psychopathologize differences between LGBTQ populations and heterosexual and cisgender populations [6, 28, 29] has reinforced the framing of LGBTQ health as the inability to maintain health at the level of the individual. Based on this framing of LGBTQ health, much health research has tended to focus on risks for poor health outcomes among LGBTQ populations, particularly rates of STI and HIV infection, smoking, obesity, depression, and suicidal ideation [30–32]. Although early health research played an important role in identifying, mitigating, and treating poor health outcomes among LGBTQ populations as something more than an individual deficit, it also served to create negative perceptions of LGBTQ health and obscured the ways in which these populations maintain their health. As such, it is necessary to shift away from deficit-focused heath research toward strengths-based perspectives that take a more holistic approach to understanding LGBTQ health across the life course [33–36]. Strengths-based perspectives do not ignore health risks and challenges but rather focus on the positive resources available to address these risks and challenges [37]. Improving cultural competence within health care systems, policies, and services in Nova Scotia requires acknowledging, rendering visible, and appropriately measuring the determinants of LGBTQ health and wellness across the life course [38, 39].

### Purpose

The purpose of this paper is to offer an overview of the findings of a scoping review and community consultations aimed at developing strengths-based approaches to understanding LGBTQ pathways to health in Nova Scotia. The scoping review and community consultations are nested within a larger program of research aimed at rendering visible the health needs, outcomes, and lived experiences of LGBTQ populations in Nova Scotia in an effort to improve access to, and the provision of, evidence-based, culturally competent health care for these populations.

## Methods

The research described in this paper is informed by the central tenets of community-based participatory research. Community-based participatory research involves “individuals and communities affected by the research in all aspects of the research process, reciprocal learning from the expertise of the members, shared decision-making, and mutual ownership of the processes and products of the research” (Van Wagenen et al., [40], p. 4). In this regard, we sought to include LGBTQ populations and other stakeholders, such as health care providers, health researchers, and policy makers, in every stage of this research.

In an effort to gain a clearer understanding of the existing health-focused LGBTQ literature, we conducted a scoping review using the methodology proposed by Arksey and O’Malley [41]. The purpose of our scoping review was to explore the academic, peer-reviewed health research literature specifically for strengths-based approaches to understanding LGBTQ health. In accordance with community-based participatory research methodology, we consulted with a community advisory committee comprised of LGBTQ community members, representatives from LGBTQ organizations, LGBTQ health researchers, and a health reference librarian to determine the search terms for our scoping review (see Appendix 1). We then conducted a search of five key databases containing academic, peer-reviewed journals using these search terms. Our initial search yielded a total of 1855 de-duplicated results, of which 105 articles met the inclusion criteria. Given that the health sector tends to draw on peer-reviewed evidence to inform best practice for clinical care and related health practice guidelines, only articles published in peer-reviewed academic journals that discussed research from strengths-based or health promotion perspectives were eligible for inclusion (see Appendix 2). As such, studies that relied primarily on a health deficit model or risk assessment approach to studying LGBTQ health were excluded. We also included studies that presented alternative analytical and methodological frameworks such as needs assessments, which can help challenge heteronormative and cisnormative approaches to LGBTQ health by allowing LGBTQ populations to identify their own health needs. To ensure that the included studies are relevant to the context of Nova Scotia and to the Canadian health care system, only studies published in English and conducted in Canada, the United States, the United Kingdom, Australia, or New Zealand were considered for inclusion. The scoping review was conducted in October 2014 and only papers published by that time were considered. We did not, however, limit our findings to a particular start date. To ensure rigour, an inter-rater reliability approach was used in the inclusion process whereby both the research assistant and principal investigator reviewed all articles flagged for possible inclusion. Articles selected for inclusion were read and thematically mapped by research question and methodological approach for consideration for health research and promoting LGBTQ health and wellness in the context of Nova Scotia.

Given the importance of engaging with LGBTQ populations, community consultations were undertaken following the completion of our scoping review to discuss the findings and their relevance in the context of Nova Scotia. We conducted two community consultations with LGBTQ populations and health service providers in Truro and in Halifax. Participants were recruited through word of mouth and through existing community networks in both urban and rural Nova Scotia. In total, there were twenty participants, six of whom attended the rural consultation in Truro and fourteen of whom attended the urban consultation in Halifax. Participants ranged in age from mid-20s to late 60s and the majority identified as white. Prior to data collection, ethics approval was provided by the Dalhousie University research ethics board (REB #2014-3291) and informed consent was obtained from all participants. All data were audio recorded with permission, transcribed verbatim, and analyzed for key emergent themes. In addition to sharing the findings of the scoping review, the overarching purpose of these consultations was to discuss strategies for conducting strengths-based research on LGBTQ health needs, outcomes, and experiences in Nova Scotia. The community consultation transcripts were analyzed and coded for emergent themes.

## Results

### Scoping review findings

Overall, the findings of our scoping review confirmed that the majority of LGBTQ health research conducted to date has largely remained focused on risks and deficits, underscoring the need to shift towards strengths-based approaches. It is also noteworthy that very few of the studies that met our inclusion criteria were conducted in Canada (*n* = 16) or were conducted in multiple countries but included populations in Canada (*n* = 3) (see Appendix 3) and only one study specifically included LGBTQ populations in Nova Scotia. This finding further illustrates the need for strengths-based research focused on the health needs and experiences of LGBTQ populations in Nova Scotia. The included studies also featured a range of study populations and terminology (see Appendix 4). While some studies focused on LGBTQ populations in general, others focused on specific subpopulations. Notable subpopulations included youth (*n* = 26), older adults (*n* = 14), and people of colour (*n* = 14). Although we did not limit our studies to a particular start date, the findings demonstrate that strengths-based research on LGBTQ health is becoming increasingly prevalent. Of the included studies, none were published prior to 1990, six were published between 1990 and 1999, 36 were published between 2000 and 2009, and 68 were published between 2010 and 2014 (See Appendix 5).

The articles included in our scoping review explored a wide range of protective or health promoting factors with the potential to contribute to LGBTQ health, including, for example, social support, coping skills, and positive school and/or work environments [33, 42–44]. Further, these studies used diverse tools to measure positive health factors. However, we also noted that many of the measures used were not specific to LGBTQ populations, and, as such, their appropriateness and utility as tools to measure LGBTQ health in Nova Scotia may be limited. Although the included studies employed a wide variety of different qualitative, quantitative, and mixed methods approaches, the use of online surveys was relatively common (*n* = 28). This finding is significant in that online surveys may provide participants with a greater degree of anonymity and have therefore been identified as an effective and appropriate way of reaching ‘hidden populations’, including LGBTQ populations, for research [29, 45–50]. The use of focus groups and interviews was also common and may be an important means of allowing LGBTQ populations to identify and discuss their own health needs and experiences.

Many of the studies included in the scoping review also featured an element of community involvement. While some involved a community advisory committee [51–54], others conducted their research in partnership with community-based organizations or service providers with the goal of informing the development or improvement of programs and services for LGBTQ populations [55–59]. These approaches are in keeping with the principles of community-based participatory research [40].

The concept of resilience, referring to the ability to overcome or positively adapt in the face of significant adversity, emerged as a commonly cited framework or theme but there is debate about whether this concept is inclusive of LGBTQ lived experiences [36, 43, 44]. While many of the included studies suggested that LGBTQ populations are in fact resilient [20, 34, 43, 60–63], resilience was not consistently defined or measured across these studies. In addition, there was no clear consensus on the factors that contribute to resilience among LGBTQ populations. Like health care systems and policies, resilience has historically been focused on individual level determinants of health, which has led some to characterize resilience as a set of inherent personal traits or skills [64–66]. This is particularly concerning given the ways in which the overemphasis on individual-level factors associated with health has contributed to the invisibility of LGBTQ health needs, outcomes, and experiences. As with the remainder of the measures used in the studies included in our scoping review, the majority of tools used to measure resilience were not LGBTQ-specific. There is also a need to approach resilience from an intersectional lens as it has historically been defined and framed from a Western perspective [65]. Overall, there is some uncertainty regarding the appropriateness and utility of the concept of resilience for LGBTQ health research in Nova Scotia and this knowledge gap warrants further exploration.

### Community consultations

The following section offers an overview of the key concerns raised about LGBTQ health in Nova Scotia from our community consultations. Our semi-structured focus group guide centred around core issues to emerge from our scoping review, including experiences with health care systems and services, factors seen to contribute to LGBTQ health and wellness, LGBTQ resilience, improving the cultural competence of health care systems and services, and finally, suggestions for future LGBTQ health research in Nova Scotia.

#### Negative experiences with health care systems and services

Following the scoping review, the community consultation discussions offered a rich overview of LGBTQ experiences with health care systems and services, factors that contribute to LGBTQ health and wellness, and how LGBTQ pathways to health in Nova Scotia can be improved. Unsurprisingly, many of the LGBTQ participants attending the consultations reported negative experiences with health care services in Nova Scotia. Several participants described having negative first impressions of health care settings based on their interactions with medical office assistants and heteronormative, gender-binary language on medical intake forms. In other words, intake forms and salutations required patients to select gender-congruent ‘male’ or ‘female’ categories and to select corresponding terms such as ‘Mr.’ and ‘Mrs’. As one participant explained, “I shouldn’t have to go into a doctor’s office and be like I’m probably going to get misgendered and I need to prepare myself for that and put on my armour. That shouldn’t even be happening in the first place. But it does happen and it’s my reality, and I have to deal with it”. Similarly, participants expressed concern about the challenges of communicating with health service providers and being open with them regarding their LGBTQ identity—both in terms of sexual orientation and gender identity. One participant characterized this experience as “explaining yourself over and over” when interacting with health service providers.

Participants suggested that these negative encounters serve to discourage LGBTQ populations from accessing regular check-ups and preventative care, and instead, waiting until they are ill before seeking health care services. Participants also expressed concern regarding health service providers’ lack of knowledge on LGBTQ health issues, which may lead to inappropriate advice. One participant shared the story of a friend in a rural setting whose doctor threatened to involuntarily commit them for psychiatric care based on their non-binary gender identification. This experience is supported by the findings of a previous study on lesbian and bisexual women in Nova Scotia wherein several women reported being told by a physician that their sexuality was pathological and referred to psychiatric services [25]. Another participant felt that health care providers in Nova Scotia may have lower expectations for their health outcomes and that this may lead to a lower standard of care in comparison with other provinces. These experiences were summarized by a participant who stated “I feel like I am surviving the health care system”. Thus, while the focus of the consultations was on advancing strengths-based research on LGBTQ pathways to health, it is important to acknowledge these negative experiences as they reinforce the importance of understanding and reconciling these tensions in access to and uptake of health care services and programs.

#### Factors contributing to LGBTQ health and wellness in Nova Scotia

One of the central discussion questions during the community consultations focused on the factors that are regarded as keeping LGBTQ populations in Nova Scotia well and promoting their health across the life course. Participants reflected on many different factors, ranging from the individual level to broader social and structural levels. Although some of these factors may also be determinants of health for the broader population, others are specific to LGBTQ populations. Participants listed widely recognized social determinants of health including socioeconomic status, access to housing, education, social isolation, and food security as key to promoting health. While these factors may be considered determinants of health for all populations, it is important to recognize how these factors intersect with LGBTQ identities. For example, as previously mentioned, LGBTQ populations face higher rates of homelessness and poverty than their heterosexual, cisgender age-matched peers [16–18]. These factors are also interconnected; as one participant pointed out, poverty among older LGBTQ populations may, for example, prevent individuals from engaging in social activities, thereby contributing to social isolation.

Participants identified health literacy and knowledge of one’s own health issues as important individual-level factors. Participants suggested that the ability to read and process health information has a significant impact on individuals’ awareness of their own health and wellness. While the issue of health literacy may not be unique to LGBTQ populations, they experience particular challenges in accessing appropriate and meaningful health information that speaks to their LGBTQ identities and lived experiences. Participants argued, for example, that sex education currently tends to be framed through a heteronormative and gender-binary lens, thereby limiting its utility for LGBTQ youth. Self-acceptance and levels or degrees of ‘outness’ to health service providers were also described as important health promoting factors. As one participant noted, “it took me a long time to get to that point to be able to talk openly about my own body [and] my own sex life”. Most participants reasoned that while not being ‘out’ to a health service provider can potentially have negative implications for health, it is also a necessary part of the process of accessing health care in order to negotiate personal safety in instances where there is uncertainty or lack of trust with a health care provider. Similarly, not being ‘out’ was seen as a factor preventing LGBTQ populations from accessing certain community organizations and services for fear of being identified as LGBTQ. In addition, cognitive, behavioural, and emotional personal coping strategies and self-care were viewed as key individual-level factors contributing to the health and wellness of LGBTQ populations in Nova Scotia.

As one participant cautioned, it is imperative that individual-level factors are not overemphasized:

when we take the emphasis off the system and put it on the individual, I worry a little bit about victim blaming…[if] I go to get care, there’s a 50 per cent chance that I’m going to leave worse than when I went in and that’s not my fault…We should also recognize that in acquiring those [personal coping] tools that there’s an injustice happening.

Consistent with our scoping review findings, social support was one of the most prominent determinants seen as contributing to LGBTQ health and wellness. Potential sources of social support include biological family or family of origin, family of choice, friends, and other LGBTQ community members. Community connectedness was also seen as a source of strength among participants. Participants defined community connectedness quite broadly, referencing involvement in gay-straight alliances, LGBTQ communities, sports leagues, community activities such as Pride Week, and accessing community services as potential connections. Similarly, participants suggested that for LGBTQ populations for whom religion or spirituality are important, belonging to an affirming religious or spiritual community could play a critical role in maintaining health and wellness. As one participant explained,

a lot of people who are [LGBTQ]… don’t feel right in the eyes of God. So they really kind of have to feel connected to a faith to actually feel that they are okay. And so we have a church that’s all affirming and we have a gay couple, one is the minister, and the whole church is just so supportive. They have rainbow stickers everywhere. So it’s that opportunity to start to feel a little bit more healthy within yourself, a little bit more whole within yourself, if that’s what you want to do.

Participants viewed the issue of pride in LGBTQ history as another important factor contributing to the health and wellness of LGBTQ populations in Nova Scotia. One participant shared stories of LGBTQ individuals who faced significant adversity but overcame them, demonstrating their strength and resilience. The participant argued that “we need more pride in our history. We need more pride in our people… and not just the ones that stood in front of the camera and became movie stars; the people that lived ordinary lives in rural communities, that lived, loved, and maybe died. But they lived together”. The same participant argued that sharing these stories and histories can be an important source of strength for LGBTQ populations. Similarly, participants suggested that having positive LGBTQ role models is a key factor contributing to their sense of wellness and social connectedness. Other factors included having safe and supportive work and/or school environments. In this regard, participants argued that acceptance within the community and in other environments is an important contributor to health. For example, one participant suggested that an individual’s social status within the community (in terms of recognition and respect) might impact acceptance within the community, which, in turn, might affect the likelihood that the individual will feel comfortable seeking health care services.

#### LGBTQ resilience

We also asked participants in the community consultations whether they felt that the concept of resilience was relevant to understanding the health needs, outcomes, and experiences of LGBTQ populations in Nova Scotia. Consistent with the lack of clarity on this concept in our scoping review results, participants spoke of the need for clarification on how resilience is defined. While participants generally felt that LGBTQ populations in Nova Scotia are resilient, the utility of this concept for LGBTQ health research remained unclear as participants struggled to define it, identify the factors that comprise it, and determine how it should be measured. This led one participant to suggest that future research should ask LGBTQ populations how they perceive their own resilience and how they would compare it to the resilience of others. Participants also discussed the need to consider measuring whether and how resilience changes over time, depending on the complex interactions of determinants of health. In terms of the determinants contributing to resilience among LGBTQ populations, participants repeated many of the same modifiable and non-modifiable determinants discussed above, including social support, pride, self-acceptance, community connectedness, and personal coping skills.

#### Improving the cultural competence of health care systems and services in Nova Scotia

In addition to determinants that contribute to promoting the health and wellness of LGBTQ populations in Nova Scotia, participants also discussed ways in which the cultural competence of health care systems and services in Nova Scotia could be improved. One of the key areas of improvement noted was making health care environments safer and more inclusive and welcoming for LGBTQ populations. Participants argued that making small changes within health and social systems such as removing heteronormative and gender-binary language from intake forms and posting visible symbols like a pride flag or an LGBTQ ally card would contribute to improving pathways to health for LGBTQ populations. As one participant explained:

if you change spaces then you could change who accesses the spaces…If I walk into a space where I see a poster on the wall where my identity is reflected, and I see a tick box on a form and know that that healthcare provider expects me in the room, then I’m more likely to access those services again.

Additionally, education and training for health care providers on how to provide culturally competent health care services for LGBTQ populations was seen as a major area for improvement. A nurse attending one of the consultations stated that, in her experience, nurses are not taught “how to make an equitable presentation for an experience in health care whatsoever. It’s just not there. We might be given one session one afternoon in our undergrad, and that’s it. And this was 2 years ago when I graduated”. This feeling echoes the views of physicians interviewed in a previous study on queer and trans women’s health care in Nova Scotia who felt that they lacked knowledge, particularly with regards to providing care for trans populations [10]. Another participant felt that the only way to ensure positive experiences of ‘coming out’ to health service providers is through additional education and training. This finding is supported by the conclusions of a previous study that found that nurses in Nova Scotia “take a ‘don’t ask, don’t tell’ approach, trusting that quality care can be provided without acknowledging LGBTQ identities and that the ways in which marginalization and oppression may shape LGBTQ patients’ health and health care” (Beagan et al. p.60 [67]). Beyond improving communication between health service providers and LGBTQ populations, educating health service providers on LGBTQ-specific health needs and issues was also seen as critically important. Overall, participants felt that education related to culturally competent care is essential for all individuals working in health care services, including medical office administrators, who are often the first people that patients interact with. One participant suggested that efforts could be made to increase the number of LGBTQ individuals interested in undertaking training to become health care providers in Nova Scotia and to offer them support for their training.

Participants argued that advocacy plays a significant role in improving pathways to health for LGBTQ populations in Nova Scotia. One participant stated that knowing their rights as a patient, such as the right to bring a friend along to an appointment, to record appointments, and to pursue formal resolution if something goes wrong, would have made them less likely to experience discrimination. Further, participants argued that having LGBTQ patient advocates who could assist LGBTQ populations in navigating health care systems is an important means of improving LGBTQ pathways to health in Nova Scotia.

Participants also shared their views on the norms that should be central in health care services and systems in Nova Scotia. For example, one participant argued that “continuity of care, meaning that you have access to a healthcare provider that you know and [that] care is personalized” is critical. Participants discussed the notion of informed consent in health care and the importance of making sure that patients have all of the necessary information to make informed decisions regarding their own health and wellness. Finally, participants viewed the notion that patients’ decisions will be supported by their health service providers as being critical in improving health care services and systems for LGBTQ populations in Nova Scotia.

#### Suggestions for future LGBTQ health research in Nova Scotia

Participants in the community consultations also identified a number of key questions for future strengths-based, health promotion research on understanding LGBTQ health in Nova Scotia. Several research questions centred on health care experiences and access. These questions included “have you ever had a positive [or inclusive] interaction with a health care provider?”, “what did that look like?”, and “how did that make a difference?”. Other participants noted that having access to a doctor in Nova Scotia can be a challenge and as such, it is important to ask whether LGBTQ populations have access to a doctor and whether they have a choice of doctors. Participants suggested that LGBTQ populations might seek health care services from providers other than their doctor, such as community nurses and teen health nurses, and that research should explore which health care provider they choose to see first and why. Participants also felt that it is important to ask LGBTQ populations whether they feel that their health service providers are LGBTQ-friendly and knowledgeable of LGBTQ health issues.

Based on the factors discussed above, participants suggested a number of key issues related to how LGBTQ populations in Nova Scotia maintain and improve their own health and wellness across the life course. For example, participants felt that it was important to ask whether LGBTQ populations are ‘out’ at work and/or school, whether these are positive environments, and if so, what factors contribute to making these environments positive. With respect to personal coping skills, participants argued that it is imperative to ascertain not only whether an individual possesses coping skills, but also how effective they are, how diverse their coping toolkit is, and whether they have the ability to develop new coping skills. Participants suggested that it is important to determine the number of support people that an LGBTQ individual has, as well as the role that those people play, and how social support affects their health and wellness.

When asked to identify who should be included in future health research focused on LGBTQ health, in addition to LGBTQ populations, participants suggested a wide range of health service providers, including emergency departments, medical office administrators, nurses, physical therapists, occupational therapists, teen health nurses, long-term care providers, telemedicine providers, public health policy makers, midwives, and continuing care assistants. In keeping with the emphasis on improving culturally competent responses among health service providers, participants felt that it was also important to include those responsible for educating health service providers, as well as students training to become health service providers. Finally, participants suggested including non-profit organizations that provide services for LGBTQ populations, such as shelters.

## Discussion: advancing LGBTQ health research in Nova Scotia

Based on the findings of our scoping review and community consultations, we argue that the determinants of LGBTQ health must be understood through a model that considers both individual and structural factors. For example, a lens of intersectionality acknowledges that health outcomes among LGBTQ populations are a result of the intersections of their LGBTQ identities with other determinants of health, including race, socioeconomic status, social exclusion, employment, etc. (see [65] for example). Further, the relationship between these factors and health outcomes is complex. For instance, while alcohol use may potentially contribute to negative health outcomes, it may also mitigate social isolation by allowing individuals to overcome social anxiety. Perhaps most importantly, rather than focusing on individual-level factors such as individual behaviour, it is important to consider how structural factors shape and influence individual risks for negative health outcomes. Similarly, rather than focusing on developing personal coping skills, there is a need to address social and structural factors such as homophobic and transphobic stigma and discrimination, particularly within health care systems, that may necessitate the use of personal coping skills.

### Implications for LGBTQ health research

Efforts to better understand the complex pathways to health among LGBTQ populations in Nova Scotia should include collecting additional data on the health needs, outcomes, and lived experiences of LGBTQ populations in Nova Scotia. The purpose of our scoping review and the community consultations described in this paper was to help inform future LGBTQ health research by exploring knowledge gaps in relation to how to understand LGBTQ health in Nova Scotia from a strengths-based perspective. Although capturing data on negative health outcomes and experiences of LGBTQ populations plays an important role in identifying, mitigating, and treating health issues, future health promotion research on LGBTQ health in Nova Scotia should also capture the ways in which LGBTQ populations maintain and improve their own health and wellness across the life course.

The strengths-based studies in our scoping review and the community consultations data provide important insights into the factors that potentially promote the health of LGBTQ populations in Nova Scotia. These strength-based determinants range from the individual level to the structural and social levels. In particular, the importance of personal coping skills, social support networks, and community connectedness were frequently cited in the scoping review and consultations as important determinants of LGBTQ health. Future health promotion research on LGBTQ populations in Nova Scotia should investigate the presence and significance of these factors and the potential for health promotion interventions to build on these strengths. At the structural level, supportive work and school environments, accepting communities, and safe, inclusive, and welcoming health care spaces were considered to have a major impact on LGBTQ pathways to health. Additional research on these structural factors in the context of Nova Scotia could contribute to policy changes that could have positive impacts on LGBTQ health outcomes. The utility of resilience as a strengths-based conceptual framework for understanding and measuring LGBTQ health in Nova Scotia also warrants further exploration. Finally, given that we were only able to conduct community consultations in two regions, there is a need for additional research on LGBTQ health in Nova Scotia that captures the perspectives of LGBTQ populations across the province.

### Limitations

Although our scoping review and the community consultations provide important information for conducting strengths-based LGBTQ health research, there are several limitations to note. The scoping review only included peer-reviewed, academic articles published in English and in academic journals and, as such, may not reflect the perspectives of non-peer reviewed or grey literature. Further, given the diversity of identities and terms related to LGBTQ populations (see Appendix 4), there may be identities or populations that were not adequately captured by the search terms, such as men who have sex with men (MSM) but do not identify as gay or bisexual, for example. While scoping reviews are a useful approach to retrieving literature related to a specific topic of interest and identifying gaps in the existing literature, they do not assess the quality of the evidence or synthesize the findings presented in the retrieved literature in the way that systematic reviews do [41]. As such, future research on this topic should consider including systematic reviews which provide a more rigorous methodology. In addition, the community consultations were limited by time and budgetary constraints which only allowed for two consultations, one in rural and one in urban Nova Scotia. Moreover, although we sought to make the consultations safe, inclusive, and respectful spaces, we invited both LGBTQ populations and health service providers to attend. This may have deterred LGBTQ individuals who have had negative health care experiences from attending. While we have highlighted the importance of intersectionality in LGBTQ health research, the majority of our community consultation participants identified as white and, as such, do not necessarily represent the diversity of LGBTQ populations in Nova Scotia. Future research should consider using alternative recruitment strategies that may result in greater diversity among participants.

## Conclusion

As the findings from our scoping review and community consultations demonstrate, there is an urgent need to conduct health research on the unique health needs, lived experiences, and outcomes of LGBTQ populations in Nova Scotia to ensure that current health policies, programs and services are responsive to these populations. Given the historical emphasis on negative health outcomes among LGBTQ populations, it is important that future health research be conducted from an intersectional, strength-based perspective in an effort to highlight not only the health risks and challenges experienced by LGBTQ populations, but also positive approaches to addressing these issues. Specifically, additional health promotion research that takes into account the social, systemic, and structural determinants of LGBTQ health is warranted.

## Appendix 1

**Table 1:** Search terms used in scoping review

Concept 1: LGBTQ identity	Concept 2: health	Concept 3: measurement
Two spirit	Resilienc*	Data collection
Lgb*	Protective factor*	Survey*
Gender minorit*	Health promot*	Model*
Sexual minorit*	Health protect*	Framework*
Trans sexual*	Life course*	Measure*
Trans gender*	Harm reduction	Tool*
Gender identit*	Health predict*	Assess*
Gender varian*	Social determinants of health	Epidemiology
Genderqueer*	Health disparities	Module
Queer*	Health status	Evaluat*
Gay*		
Lesbian*		
Bisexual*		
Transgender*		
Transsexual*		
Homosexual*		
Intersex*		

## Appendix 2

**Table 2:** Inclusion/Exclusion criteria for scoping review

Inclusion	Exclusion
Published in English	Published in language other than English
Peer-reviewed	Non peer-reviewed
Academic journal article	Book, dissertation, conference abstract, etc.
Primary study	Not a primary study
Study conducted in US, UK, Australia, New Zealand or Canada	Study conducted in country other than US, UK, Australia, New Zealand or Canada
Approaches LGBTQ health from a strengths-based or health promotion perspective	Approaches LGBTQ health from a deficit-based or risk-focused perspective

Time Frame: The scoping review was conducted in October 2014. All included results were published before then. We did not limit our search using a start year

## Appendix 3

**Table 3:** Study locations

Country	# of studies
United States	70
United Kingdom	3
Canada	16
New Zealand	5
Australia	6
Multiple countries	5

## Appendix 4

**Table 4:** Study populations

Study population	# of studies
Gay men	16
Gay and bisexual men or men who have sex with men (MSM)	12
Bisexual individuals	2
Lesbian women	10
Lesbian and bisexual women	2
Transgender individuals	16
Gay and lesbian individuals	4
Lesbian, gay, and bisexual individuals (LGB)	9
Lesbian, gay, bisexual, and transgender individuals (LGBT)	15
Lesbian, gay, bisexual, transgender, and queer individuals (LGBTQ)	7
Sexual minority individuals	5
Transgender, queer, and questioning individuals (TQQ)	1
Gay, lesbian, bisexual, transgender, and intersex individuals (GLBTI)	2
Gay and bisexual men and male-to-female (MTF) transgender individuals	1
Lesbian, gay, bisexual, and queer individuals	2
LGBTQ women	1

## Appendix 5

**Table 5:** Historical trends in included studies

Date of publication	Number of studies
Before 1990	0
1990–1999	6
2000–2009	31
2010–2014	68

## Abbreviations

COPD: Chronic obstructive pulmonary disease
HIV: Human immunodeficiency virus
LGBTQ: Lesbian, gay, bisexual, transgender and queer/questioning
PHAC: Public Health Agency of Canada
STI: Sexually transmitted infection
WHO: World Health Organization
